# 

**Published:** 1989-11

**Authors:** 


**Editor**
Mr M. G. Wilson
*40 Coombe Lane, Bristol BS9 2BJ*
*Tel: 0272-682320*

**Editorial Committee**
Dr Paul Goddard (Assistant Editor)
Mr B. P. Jones (Secretary)
Dr J. L. Burton

**Correspondents**
Dr A. E. Adam (Taunton)
Mr D. C. C. Bartolo
Mr M. L. Crosfil (Penzance)
Dr J. D. Davies
Dr J. Jancar
Professor D. J. Pereira Gray (Exeter)
Dr H. G. Mather
Professor G. M. Stirratt
Dr J. L. G. Thomson
Dr M. J. Whitfield
Professor G. K. Wilcock

**BRISTOL MEDICO-CHIRURGICAL SOCIETY**

Mr C. R. Feneley (President)

Mr R. N. Baird (Secretary)
*23 Old Sneed Park, Bristol BS9 1RG*

Dr N. C. Tricks (Treasurer)
*The Mill, Wrington, Bristol*

**Published by**
**Clinical Press,**
*15 Bucklands Drive*
*Nailsea*
*Bristol*

**Typesetting by**
*Apek Typesetters*
*Avon House, Nailsea, Bristol BS19 2DJ*

**Printed by**
*Sutton Print, Paignton*

THE BRISTOL MEDICO-CHIRURGICAL JOURNAL
acknowledges with gratitude the help it receives from
THE NUFFIELD NURSING HOMES TRUST

Established 1883

### BRISTOL MEDICO-CHIRURGICAL JOURNAL

**NOVEMBER 1989**
**Volume 104(iv)**

Copyright © Clinical Press Ltd 1989.

**Contents**

89 **Editorial**

90 **Impressions**

A. John Webb

96 **Obituary—Peter Campbell Clifford**

97 **Carey Franklin Coombs 1879–1932**

Clive F. M. Weston

104 **Inguinal Hernia Waiting Lists; Medical and Financial Implications**

A. H. Davies, J. Mountfield, C. P. Armstrong

105 **A case of Self-Inflicted Penile Ulceration**

D. S. Allen

107 **From Our Correspondents**

109 **Leather**

J. M. A. Smith

112 **South West Orthopaedic Club**

Meeting at Newport, November, 1988

115 **Index for 1989**

*What I know remains unknown, unless by me to others shown.*
**Lucilius (180–102 BC)**

### THE MEDICAL JOURNAL OF THE SOUTH WEST

**NOTICE TO CONTRIBUTORS**
Contributions are invited especially from West of England authors on a variety of subjects, e.g. Original articles relating to the practice of medicine, reports on the contemporary medical scene, obituaries, historical accounts, recollections of colleagues and events worthy to be remembered and liable to be forgotten. An article of 2,000 words with 2 illustrations will occupy 2 pages and this is a suggested, though by no means an obligatory length. Articles are preferred in the form proposed by the International Committee of Medical Editors (Vancouver System)—pamphlet obtainable from Editor of BMJ., BMA. House, Tavistock Square, London, WC1H 9JR.

### INDEX to Volume 104, nos i, ii, iii and iv 1989.

Only the first two named authors to each paper are listed.

Abrams, P., 55
Absidia, bronchial, 31
Allen, D. D., 105
Artificial urinary sphincters—early experience, 55
Avon Breast screening programme, 33
Ainscow, D. A. P., 32a
Aorta, management of dissection of thoracic, 25
Are you being served?, 15
Arthroplasty of the 1st metatarsal joint with silastic spacer, 112
Asthma Nurse, benefits and costs of, 11
Austin, A. D. L., 83
Autotransfusion in aortic surgery, 26

Backache, computed tomography in the investigation of, 87
Banks, R., 32a
Bannister, G., 27
Barham, C. P., 86
Barker, R., 85
Bear, J. D., 28
Benbarka, H., 113
Bennet, G., 13
Bowel preparation for colonic resection, 85
Bloom, P. A., 23
Boggis, C. R. M., 35
Book Reviews
  Essential paediatric dermatology, Julian Verbov
  Rheumatic diseases and the heart, J. A. Cosh & J. V. Lever
Bracey, D. J., 88
Bradley, M. C., 75
Breast cancer, chemotherapy in, 85
Breast cancer, conservative management of, 24
Breast cancer, conservative surgery for, 47
Breast cancer, epidermal growth factor receptors and, 51
Breast cancer and the Radiation Oncologist, 53
Breast carcinoma and the National Screening Programme, 31
Breast cytology, intraoperative, 86
Bright, Richard—a man of many parts, 63
Britton, J. M., 113
Burton, J. L., 19, 82

C.D.H. Screening programme, 24
Carswell, F., 11, 72
Clarke, N. M. P., 29
Clay, N. R., 113
Colles fracture, treatment of unsatisfactory reduction, 113
Collins, C., 88
Conseal continent colostomy appliance, 59
Cook, D., 13
Cook, P. G., 42
Coombs, Carey Franklin, 1879–1932, 97
Creighton, S., 55
Crosfil, M., 79
Cross, M. J., 112
Cruciate ligament reconstruction with goretex, 27
Cytoguide stereotactic biopsy system, 54

Davies, A. H., 104
Davies, Z., 33
Deutscher, D., 26
Down, G., 59
Drayton, M. R., 30
Dunn, P. M., 30

Eltringham, R. J., 24
Embolectomy, which balloon catheter?, 26
Empoisoned Darts of Venus; John Warren, MD, 17

Fairclough, 27
Fairgreave, J., 26, 26
Farndon, J. R., 51, 70
Fermoral fractures, use of interlocking nails for, 113
Femoral fractures, subtrochanteric, use of dynamic hip screw, 114
Fleischl, J. M., 26
Forefoot surgery, pressure plate analysis in, 87
Forrester Wood, J., 31

Gastric asthma, 31
Gibbons, C. M. L. H., 113
Gibson, R. A., 85
Gie, G. A., 88
Gilbert, H. W., 68
Gillatt, D. A., 47
Gingell, J. C., 68
Glove perforation, a study of mechanisms, 86
Goddard, P., 42
Goodman, S., 53
Graham, G., 27
Green, T. P., 24
Guy, J. R., 17

Haematuria, Identification of source of, 32a
Hallux valgus, chielectomy for, 113
Hamilton, A. H., 72
Harvey, J., 31
Health Knowledge in Rural Practice, 83
Heather, B. P., 25
Higgs, C. M. B., 32
Hip fractures, Hansson pins for, 113
Houghton, M., 23
Hussell, J. G., 112
Hutchins, P. M., 87
Hyperthyroidism, asymptomatic and recurrent, 70
Hypoxia and the brain, 30

Impressions, 90
Inguinal hernia waiting lists, 109
Intensive Care Organisation, 24
Interphalangeal joint movements, wrist position in, 113

James, E. D., 86
Jeyasingham, J., 31
Joint replacement, antibiotics in, 28
Johnson, D. P., 87
Johnson, S. R., 113
Jones, G., 113

Knee ligament reconstructions for instability, 112
Knee, anterior cruciate ligament repair, 112
Knee, synovectomy of, 87
Keen, G., 25
Kennedy, C., 32a

Laurence, B. M., 29
Lavy, C. B. D., 113
Leaper, D., 59
Leather, 109
Levers, B. G. H., 15
Limb Salvage, 25
Looking back on 40 years of Paediatrics, 29

Lucarotti, M., 86
Lumb, G. N., 25
Lung cancer, resection in elderly patients, 31

MacGavin, C., 32
MacKenzie, C., 63
Mackie, I. G., 112
Magnetic Resonance Imaging and Carcinoma of the Breast, 42
Maheson, M., 87
Majkowski, R. S., 88, 113
Mammography, practical aspects of, 35
Maneshka, J. R., 31
May, P. C., 28
May, P., 112
May, R. E., 47
Mayo's arthroplasty, 88
Mediastinal Mass, 31
Meningococcal disease in Gloucestershire, 29
Metastatic Disease of Thoracic and lumbar spine, 32a
Morris, P., 16
Morris, R. D. A., 88
Motor cycles, deaths due to, 85
Motorbike legs, 86
Mott, Martin, 3
Mountfield, J., 104
Mulloy, E., 31
Mutation, malformation and malignancy, 3

Narcolepsy with apnoea, 32
Neonatal brain, circulatory adaptation in, 30
New dimensions in the NHS, 19
Newill, R. L. M., 86
Nicholson, S., 51

Obituary
  Peter Campbell Clifford, 96
  William H, Hylton, 67
Osteo-arthritis in young sportsmen, 27
Ophthalmic trauma, 85
Owen, J. R., 24
Oxborrow, S., 31

Patellar subluxation following knee arthroplasty, 87
Paediatric Dermatology, 32a
Palmer, J. D., 86
Pancreatic Transplantation, 16
Paracetamol, hypothermic response to, 31
Patient Management, 75
Pediculus humanus capitis in an immuno-compromised patient, 23
Penile ulceration, self inflicted, 105
Pericardial effusion, chronic, 32
Perinatal pathology, pastimes in, 30
Pelvic injuries in association with traumatic abduction of the leg, 28
Pelvic sepsis after anastomotic leak, 26
Preoperative localisation of impalpable breast lesions, 43
Prior, J., 31
Proctocolectomy, experience with restorative, 24
Prostatic symptoms, review of patients on W. L. with, 86
Psychopathology in Art—meditation on Francis Bacon, 80

Ricketts, J. W. S., 86
Reidy, J. F., 43
Renal function in the newborn, 30
Reynolds, I. S. R., 27
Reynolds, S., 32a
Robinson, E. J., 11
Ross, C. M. D., 85

Savage, R., 113
Scoliosis in Cornwall, 88
Scott, D. J. A., 28
Scully, C. M., 15
Seal, P. V., 27
Sellwood, R. B., 87
Sexual Offenders, alternative approach to, 68
Shakespeare—a new discovery, 79
Siddorn, D., 31
Silver, 31
Smith, E. J., 112
Smith, J. M. A., 109
Smith, W. H., 87
South West Orthopaedic Club
Meeting in Taunton, November 1987, 27
Meeting in Truro, April 1988, 87
Meeting in Newport, November 1988, 112
South West Paediatric Club
Meeting in Bristol, October 1987, 29
Spinal anaesthesia in total hip arthroplasty, 88
Splendid but hopeless, 82
Stuart, J. M., 29
Surgical Club of S.W. England
Meeting at Cheltenham, November 1987, 24
Meeting at Barnstaple, October 1988, 85
Susan Britton Wills Unit, Bristol General Hospital, 13

Tasker, T. B. P., 24
Thomson, W. H. F., 24
Thoracic Outlet Compression and the Rhoos Operation, 26
Togantzi, M., 112
Tracheobronchial foreign bodies, 72

Ultrasound, diagnostic in urology, 25
Ultrasound examination of the infant hip, 29

Vesey, S. G., 25

Webb, A. J., 31, 90
West Country Chest Society
Meeting in Exeter, March 1988, 31
Weston, C. F. M., 97
White Paper and the new NHS, editorial on, 89
Whitfield, M. J., 75
Wilkins, B. H., 30
Wilson, T., 26
Wilson, M. G., 80, 89
Wisheart, J. D., 72
Woodwards, R., 113
Wound infection in elective orthopaedic surgery, 27

